# AMOTL2 inhibits lymph node metastasis in gastric cancer by suppressing TGF-β/Smad signaling and is destabilized by the E3 ubiquitin ligase SMURF1

**DOI:** 10.7150/ijbs.135795

**Published:** 2026-08-11

**Authors:** Huizhen Wang, Bo Yang, Changqing Lin, Yigao Wang, Yida Lu, Jing Li, Jianhui Li, Kexun Yu, Mingliang Wang, Pengpeng Liu, Yuexiang Wang, Yongxiang Li

**Affiliations:** 1 Department of General Surgery, the First Affiliated Hospital of Anhui Medical University, Hefei, 230022, China,; 2 Department of General Surgery, the Second Affiliated Hospital of Anhui Medical University, 678 Furong Road, Hefei, 230601, China,; 3 Department of General Surgery, Hunan Provincial People's Hospital (the First Affiliated Hospital of Hunan Normal University), Changsha, 410005, China,; 4 Department of Gastrointestinal Surgery, Henan Provincial People's Hospital, Zhengzhou, 450003, China,; 5 Shanghai Institute of Nutrition and Health, Chinese Academy of Sciences, Shanghai 200031, China.

**Keywords:** Gastric cancer, AMOTL2, SMURF1, TGF-β/Smad pathway

## Abstract

Angiomotin-like protein 2 (AMOTL2) regulates cell polarity and cytoskeletal regulation, but its role in gastric cancer (GC) remains undefined. Here, we show that AMOTL2 is frequently downregulated in GC tissues and cell lines, and its low expression is associated with deeper tumor invasion, lymph node metastasis, and shorter overall survival. Gain- and loss-of-function assays demonstrated that AMOTL2 suppresses GC cell proliferation, migration, and invasion. AMOTL2 overexpression also impaired endothelial and lymphatic-endothelial tube formation and reduced the expression of angiogenic and lymphangiogenic factors. In the subcutaneous, orthotopic, and popliteal lymph node metastasis models, AMOTL2 inhibited tumor growth and lymphatic dissemination. Mechanistically, AMOTL2 suppressed TGF-β/Smad signaling, as evidenced by reduced BMP2 and TGF-β1 expression, decreased Smad1/5/9 and Smad2/3 phosphorylation, and impaired Smads nuclear accumulation. Notably, pharmacological Smad1/5/9 activation by SB4 partially reversed the suppressive phenotypes of AMOTL2 *in vitro* and *in vivo*. Co-immunoprecipitation, GST pull-down, and ubiquitination assays identified SMURF1 as an AMOTL2-interacting E3 ubiquitin ligase that binds through its WW domains and promotes AMOTL2 ubiquitination and proteasomal degradation. AMOTL2 restoration counteracted SMURF1-driven malignant phenotypes. Collectively, these findings define AMOTL2 as a potential suppressor of GC progression that is destabilized by SMURF1, suggesting that the SMURF1-AMOTL2-Smad signaling axis may contribute to GC progression and lymphatic dissemination, representing a candidate therapeutic target that warrants further validation.

## Introduction

Angiomotin (AMOT) was originally identified as an angiostatin-binding protein mediating anti-angiogenic effects 1. Subsequently, AMOTL1 and AMOTL2 were identified as related members of the angiomotin family, which is characterized by conserved coiled-coil domains and C-terminal PDZ-binding motifs 2-4. AMOTL2 functions as a cytoplasmic scaffold that restrains the Hippo pathway effectors YAP and TAZ by sequestering them in the cytoplasm 5. It also binds AKT and impairs its membrane translocation, and activates LATS2-dependent YAP phosphorylation 6, 7. AMOTL2 activity is fine-tuned by post-translational modifications: mTORC2-mediated phosphorylation at Ser760 disrupts the interaction between AMOTL2 and YAP and attenuates its growth-suppressive activity 8. In contrast, mono-ubiquitination at K347 and K408 enhances LATS binding and YAP phosphorylation 9, 10, whereas USP9X-mediated deubiquitination at K437 stabilizes AMOTL2 and strengthens YAP/TAZ restraint 9, 11. Beyond the Hippo signaling, AMOTL2 also contributes to cytoskeletal organization through interaction with LL5β 12.

The biological functions of AMOTL2 are context dependent. Tumor-suppressive effects have been described in lung adenocarcinoma, non-small-cell lung cancer, medulloblastoma, and glioma 13-16. In these malignancies, AMOTL2 inhibits cell growth and motility through AKT, JUN, or Wnt/β-catenin signaling pathways. Conversely, AMOTL2 has been reported to promote p38-dependent tumorigenicity in luminal breast cancer and to facilitate ERK-dependent endothelial cell migration and angiogenesis 17-19. AMOTL2 is also detectable in glioma stem cells, but its cellular functions in this context remain poorly understood 20. Similarly, its role in gastric cancer (GC) has not been explored to date.

SMURF1 is a HECT-type E3 ubiquitin ligase containing a C2 domain, two WW domains, and a catalytic HECT domain. It is a well-established regulator of TGF-β/BMP signaling and can target receptor-regulated Smads (Smad1/5/9), inhibitory Smads, and TGF-β receptor complexes for ubiquitin-dependent turnover 21-24. SMURF1 is frequently increased in various cancers and promotes malignant progression through ubiquitination of substrates including Kindlin-2, ARHGAP26, RHOA, and FOXA2 25-35. However, context-dependent tumor-suppressive effects have also been reported, wherein SMURF1 exerts suppressive function in hepatocellular carcinoma through promoting degradation of several pro-tumorigenic substrates TRIB2 and MCAM 36-38. In GC tissues, SMURF1 is frequently increased, and associated with shorter overall survival 25, 27, 39. Its knockdown suppresses proliferation, migration, tumor growth, and liver metastasis, potentially through PI3K/AKT signaling 25. Moreover, SMURF1 is negatively regulated by tumor-suppressive microRNAs, including miR-1254 and miR-424 27, 39, 40. Unexpectedly, SMURF1 also exerts as a suppressive factor in GC by degradation of MEKK2, leading to suppression of MEK/ERK signaling, this process was promoted by Stk38 and repressed by Kir2.1 41, 42.

Accumulating evidence indicates that multiple Smad proteins, including Smad2/3 and Smad1/5/9, have been implicated in GC progression. The TGF-β2/TGF-βR/Smad2/3 axis can upregulate NDRG1 and promote cell migration and epithelial-mesenchymal transition (EMT) 43, whereas METTL3-dependent regulation of Smad3 expression can enhance pathway activation 44. Within the tumor microenvironment, reciprocal TGF-β1/Smad2 signaling between GC cells and bone marrow mesenchymal stem cells (BMSCs) can promote cancer-associated fibroblast differentiation and tumor-cell EMT 45. BMP-Smad signaling also contributes to gastric tumorigenesis: mTOR can potentiate BMP-Smad1 activity 46, GLIS3 has been linked to the TGF-β1/TGF-βR1/Smad1/5 pathway 47, and BMP4 activates Smad1/5/9-ID1 to promote EMT and metastasis 48. Collectively, these observations suggest that both Smad2/3 and Smad1/5/9 pathways promote malignancy via distinct molecular axes, reflecting their functional complexity and context-dependent roles in gastric tumorigenesis.

In this study, we investigated the clinical relevance and functional role of AMOTL2 in GC, with particular emphasis on tumor progression, lymphatic dissemination, and TGF-β/Smad signaling. We also sought to identify an E3 ubiquitin ligase that controls AMOTL2 protein stability. To this end, clinical tissue analyses were integrated with gain- and loss-of-function experiments, endothelial and lymphatic-endothelial assays, protein-interaction studies, and several xenograft models. The study was designed to explore the putative AMOTL2-Smad regulatory axis and to preliminarily assess its prognostic and therapeutic relevance in GC, findings that require further validation.

## Materials and methods

### Cell culture

Gastric epithelial cells GES-1 and GC cell lines (AGS, SGC7901, MGC803, HGC27, SNU-1, MKN-45, MKN-74, and NCI-N87) were purchased from Shanghai Cell Bank, Chinese Academy of Sciences. Cell identity was authenticated by short tandem repeat profiling. Cells were cultured in RPMI 1640 medium (10-040-CVR, Corning, USA) supplemented with 10% fetal bovine serum (FBS, FB25015, Clark Bioscience, USA) and 1% penicillin/streptomycin solution (P/S, SV30010, HyClone, USA). Human endothelial cell lines (HUVECs, CL-0675, Procell, China; HLECs, VCH00407, ViCell, China) were maintained in endothelial cell medium (1001, ScienCell, USA) supplemented with FBS (0025, ScienCell, USA), endothelial cell growth supplement (ECGS, 1052, ScienCell, USA), and P/S (0503, ScienCell, USA). All cells were maintained at 37°C in a cell incubator containing 5% CO_2_ (HERACELL 150i, Thermo, USA).

### Patient information

Two tissue microarrays (TMAs) described in our previous studies were analyzed 49, 50. The first TMA comprised samples from 129 GC patients with pathologically confirmed GC who underwent radical gastrectomy at the First Affiliated Hospital of Anhui Medical University (December 2006-May 2008) 49. The second TMA comprised 107 GC samples collected between October 2012 and December 2013 50. None of the patients received preoperative chemotherapy or radiotherapy. Tumors were staged according to the American Joint Committee on Cancer criteria. Clinicopathological characteristics are summarized in **Table 1** and** Supplementary Table S1**. In addition, 28 paired fresh GC and adjacent non-tumor tissues were collected for RT-qPCR. This study was approved by the Ethics Committee of the First Affiliated Hospital of Anhui Medical University (2023450), was conducted in accordance with the Declaration of Helsinki, and included written informed consent from all participants.

### Immunohistochemistry assay

AMOTL2 expression was assessed by immunohistochemistry (IHC) in the first GC TMA. Staining was independently evaluated by two pathologists blinded to clinical data. The IHC scores were calculated by multiplying the staining intensity score (0: negative; 1: light yellow; 2: brownish yellow; and 3: dark brown) by the percentage of positive cells (0: 0%; 1: 1%-25%; 2: 26%-50%; 3: 51%-75%; and 4: 76%-100%). Scores were defined as high (≥ 6) or low (0-4); a score of 5 cannot arise from this scoring system. Correlations with clinicopathological parameters and overall survival were analyzed as described below using GraphPad Prism. SMURF1 expression was evaluated in the second TMA using the same scoring system.

### RT-qPCR assay

Total RNA was extracted using TRIzol (15596018, Thermo, USA) and quantified with a BioPhotometer® D30 (Eppendorf, Germany). cDNA was synthesized using cDNA Synthesis SuperMix (11141ES10, Yeasen, China), and real-time quantitative PCR was performed with SYBR Green Master Mix (11202ES03, Yeasen, China) on a QuantStudio^TM^ 3 system (Thermo, USA). Relative expression was calculated using the 2^-ΔΔCt^ method using GAPDH as an internal control. Primer sequences were as follows: AMOTL2-F: 5'-GGGAGCAGAAGTATTTGGAGGAACG-3'; AMOTL2-R: 5'-GAATGTCGGATGAGAGTGGTGTCAC-3'; GAPDH-F: 5'-CTCTGCTCCTCCTGTTCGAC-3'; GAPDH-R: 5'-ACGACCAAATCCGTTGACTC-3'.

### Plasmid construction and viral infection

Full-length human AMOTL2 (NM_016201) was amplified and cloned into the Ubi-MCS-SV40-Neomycin vector through the AgeI and NheI sites. For knockdown, shRNA-1 (5'-GTTGAGTGAACGGCTCCTTCA-3') and shRNA-3 (5'-AGGAGATGGAAAGCAGGTTAA-3') were inserted into pLKO.1-TRC vector. Full-length human SMURF1 (NM_020429.3) was cloned into the pLVX-puro vector through the XhoI and XbaI sites. All constructs were verified by DNA sequencing. Lentivirus particles were produced by co-transfecting HEK293T cells with the target vector, psPAX2, and pMD2.G using PEI 40K (G1802, Servicebio, China). The PEI-to-DNA ratio was 4 μL:1 μg, and the mass ratio of psPAX2:pMD2.G:target plasmid was 1:1.5:2. Virus supernatants were collected 48-72 h post-transfection, filtered, and used to infect AGS and SGC7901 cells in the presence of polybrene (C0351, Beyotime, China). Stable cells were selected with G418 (MA0321, Meilun Bio, China) or puromycin (ST551, Beyotime, China) and maintained under the corresponding selection thereafter.

### EdU assay

DNA replication was assessed using the EdU Cell Proliferation Kit with Alexa Fluor 594 (C0078S, Beyotime, China). AMOTL2-overexpressing cells, AMOTL2 knockdown cells, and their corresponding controls were seeded on glass coverslips (BS-14-RC, Biosharp, China) in 12-well plates. On the following day, cells were incubated with 10 μM EdU for 2 h, then exposed to the click additive solution for 30 min at room temperature in the dark. Nuclei were counterstained with Hoechst 33342 (1:1000 in PBS) for 10 min. Images were captured randomly using a DMi1 microscope (Leica, Germany), and EdU-positive cells were analyzed using ImageJ software.

### Cell migration and invasion assay

Cell invasion and migration were assessed in Transwell inserts with 8.0-µm-pore (3422, Corning, USA). Cells were serum-starved for 24 h before analysis. For invasion assays, the upper chamber was pre-coated with 100 μL of 1 mg/mL Matrigel (1:10 in serum-free medium) (356234, Corning, USA) and incubated for 5 h; this coating step was omitted for migration assays. AGS cells (8 × 10^4^) or SGC7901 cells (1 × 10^5^) were seeded into the upper chamber in serum-free medium, and 650 μL medium with 20% FBS was added to the lower chamber. After 22 h for migration or 28 h for invasion, non-migrated cells were removed from the upper surface. Cells on the lower surface were fixed with 4% paraformaldehyde, stained with 1% crystal violet. Images were captured using a microscope and analyzed using ImageJ.

### Wound healing assay

AGS or SGC7901 cells were seeded in 6-well plates and grown to 100% confluence. A linear scratch was generated using a sterile 10 μL pipette tip, and detached cells were removed by PBS washing. Cells were cultured in serum-free medium and images were captured at 0, 24, and 48 h using a Celldiscover 7 microscope (Zeiss, Germany). Wound closure was quantified using ImageJ.

### Matrigel-induced tube formation assay

To evaluate the effect of tumor-cell AMOTL2 on endothelial tube formation, HUVECs or HLECs were co-cultured with SGC7901-AMOTL2 or control cells in 0.4-µm-pore Transwell chambers (3450, Corning, USA). For tube formation, 48-well plates were pre-coated with 50 μL Matrigel per well and incubated at 37 °C for 60 min. Endothelial cells were detached with 0.25% trypsin/EDTA (SH30042.1, HyClone, USA), centrifuged, resuspended in serum-free medium, seeded onto polymerized Matrigel at 6 × 10⁴ cells in 200 μL per well, and incubated for 5 h. Random fields were imaged using a Celldiscover 7 microscope and quantified with ImageJ.

### Periodic acid-Schiff (PAS) and CD31 double staining assay

Paraffin-embedded tumor sections were incubated with anti-CD31 antibody (1:50; ab28364, Abcam, UK), followed by secondary antibody (PV-6000, Zsbio, China) for 20 min at 37 °C. Immunoreactivity was visualized with DAB (ZLI-9018, Zsbio, China). Sections were then stained with a PAS Stain Kit (G1280, Solarbio, China), including incubation with Schiff reagent for 10-15 min and hematoxylin counterstaining (Baso, China). Slides were dehydrated, cleared, mounted, cover-slipped, and scanned using CaseViewer (3DHISTECH Ltd, Hungary). CD31-negative/PAS-positive channels were interpreted as vasculogenic mimicry structures, whereas CD31-positive channels were considered endothelial vessels.

### Western blot analysis

Cells were lysed with M-PER buffer (78501, Thermo, USA) with protease and phosphatase inhibitors (K1007, K1015, APExBIO, USA) on ice for 30 min. Nuclear and cytoplasmic fractions were separated using a Nuclear and Cytoplasmic Protein Extraction Kit (P0028, Beyotime, China). After electrophoretic separation and transfer, membranes were blocked with 5% non-fat milk in TBST for 1 h at room temperature, then incubated overnight at 4°C with the primary antibodies listed in **Supplementary Table S3**. After three TBST washes, membranes were incubated with HRP-conjugated secondary antibodies (Sangon Biotech, China) for 1 h at room temperature. Signals were visualized using a 5200 Multi imaging system (Tanon, China) with an ECL kit (D045-2, Bridgen, China), and quantified using ImageJ.

### Co-immunoprecipitation and mass spectrometry (MS)

To identification and validation of AMOTL2-interacting proteins, SGC7901-AMOTL2 and control cells were lysed in M-PER buffer with inhibitors. Lysates were centrifuged at 12,000 × g for 10 min at 4 °C, and 50 μL of each supernatant was saved as input. The remaining lysate was incubated overnight at 4 °C with protein A/G magnetic beads (B23201, Selleck, China) pre-bound to mouse IgG (sc-2025, Santa Cruz, USA), anti-HA (AE008, ABclonal, China), or anti-Flag antibody (AE005, ABclonal, China). After washing beads with TBST, the bound proteins were eluted with M-PER buffer, and boiled in 5× loading buffer at 95 °C before western blot or MS analysis.

For MS, immunoprecipitated samples were separated by SDS-PAGE, excised, and subjected to in-gel digestion. Peptides were separated on a C18 column using an EASY-nLC™ 1200 system coupled to a Q-Exactive mass spectrometer (Thermo, USA). Spectra were searched against in a human UniProt-Homo database (v.2.4). Candidate AMOTL2-interacting proteins were identified after exclusion of proteins detected in the IgG control.

### Cell-based ubiquitination assay

HEK293T cells were co-transfected with plasmids encoding HA-tagged AMOTL2 (AMOTL2-HA), 3 × Flag tagged SMURF1 (SMURF1-3F), and His-tagged ubiquitin variants (wild-type, K6/K11/K27/K29/K33/K48/K63; each containing only the indicated lysine residue with other lysine mutated to arginine). Before collection, cells were treated with 10 μM MG132 (HY-13259, MCE, China) for 8 h. Lysates were immunoprecipitated with an anti-HA antibody, and ubiquitin signals in immunoprecipitated proteins were detected by immunoblotting with an anti-ubiquitin antibody.

### Molecular docking analysis

Structural models for AMOTL2 (Q9Y2J4) and SMURF1 (Q9HCE7) were obtained using the AlphaFold3 server (https://alphafoldserver.com/), which was also used to predict a putative AMOTL2-SMURF1 complex. Predicted models were ranked according to the platform output, with the top ten highest-ranked models were retained for further analysis. Molecular representations were generated using PyMOL (Schrödinger, LLC).

### GST pull-down assay

GST pull-down assays were performed to test whether AMOTL2 and SMURF1 interact directly *in vitro*. His-tagged AMOTL2, GST-tagged full-length SMURF1, and GST-tagged SMURF1 lacking the WW regions were purified. Purified His-AMOTL2 was incubated overnight at 4 °C with GST-SMURF1-WT, GST-SMURF1-ΔWWs, or GST control. Glutathione agarose beads (HY-K0211, MCE, China) were then added and incubated for additional 3 h at 4 °C. After extensive washing, bound proteins were eluted and analyzed by western blot using anti-His and anti-GST antibodies.

### Dual-luciferase reporter assay

18 pathway-reporter constructs were generated in the psiCHECK-2 backbone using core response elements for AP-1, E2F1, ELK1, HIF1, Myc, NF-κB, RBP-Jκ, Smad, TCF/LEF, p53, ATF2/3/4, ATF6, CREB, EGR1, NFAT, Nrf1/2, STAT1/2, and STAT3. Reporter sequences are listed in **Supplementary Table S4**. HEK293T cells were co-transfected with an AMOTL2 overexpression plasmid or empty vector and the individual reporter. After 48 h, luciferase activities were measured using the Dual-Lumi™ Luciferase Assay Kit (RG088S, Beyotime, China) on an EnSpire Multimode Plate Reader.

### Animal models

All animal experiments were approved by the Animal Ethics Committee of Anhui Medical University (LLSC20231297). Four- to five-week-old male BALB/c nude mice (GemPharmatech, China) were used.

For the subcutaneous model, mice were randomly divided into groups (n = 6 per group). SGC7901-AMOTL2 cells or control cells (5 × 10⁶ in 100 μL PBS) were injected subcutaneously into the right flank. Tumor length and width were measured every 2 days. Mice were then euthanized when tumors reached predetermined volume, and tumors were harvested and weighed.

For gastric orthotopic model, luciferase-expressing SGC7901-AMOTL2 (SGC7901-AMOTL2-Flu) cells or control cells (5 × 10⁶ in 50 μL PBS; n = 4 per group) were orthotopically injected into the gastric subserosa. Tumor growth was monitored by bioluminescence imaging (AmiX, SI Imaging, USA) after administration of D-luciferin (150 mg/kg, Biolite, China). Mice were then euthanized at the study endpoint, and tumors were collected for histologic analysis.

For the popliteal lymph node metastasis model, SGC7901-AMOTL2-Flu cells or control cells (5 × 10⁶ in 50 μL PBS) were injected into the left footpad. Primary-tumor growth and enlargement of the popliteal lymph node were monitored. Bioluminescence imaging was performed before euthanasia, after which footpad tumors and popliteal lymph nodes were dissected. In addition, to adjust for inter-animal variations in primary tumor growth, the ratio of popliteal lymph node bioluminescence to the total signal (lymph node plus footpad) was calculated and used to quantify relative lymph node metastatic index.

For pharmacologic rescue, SB4 (5 mg/kg in 100 μL 10% DMSO/90% PBS) 51, 52 was administered intraperitoneally every 2 days beginning on day 10, for a total of six doses. This regimen was applied in the subcutaneous and popliteal lymph node metastasis models.

For SMURF1 overexpression models, SGC7901-SMURF1-Flu cells or control cells were implanted subcutaneously or injected into the footpad according to the protocols described above. Tumor growth and lymph node metastasis were monitored by bioluminescence imaging. Harvested tissues were evaluated by H&E staining, IHC for Ki67, AMOTL2, or SMURF1, and CD31/PAS double staining, as indicated.

### Statistical analysis

Statistical analyses were performed using SPSS version 27.0 (IBM, USA) and GraphPad Prism (GraphPad Software, USA). All data are presented as mean ± SD from at least three independent experiments. The unpaired or paired Student's t-test were used for two-group comparisons, and two-way ANOVA was applied for cell-growth-curve analyses, as specified in the figure legends. The associations between AMOTL2 or SMURF1 expression and clinicopathological parameters were analyzed with the chi-square test. Overall survival curves were estimated by the Kaplan-Meier method and compared with the log-rank (Mantel-Cox) test. Cox proportional hazards regression models were used for univariate and multivariate survival analyses. *P* < 0.05 was considered statistically significant. Significance is denoted as *p < 0.05, **p < 0.01, ***p < 0.001, ****p < 0.0001.

## Results

### Reduced AMOTL2 expression was associated with aggressive clinicopathological features and shorter survival

**Figure 1:**
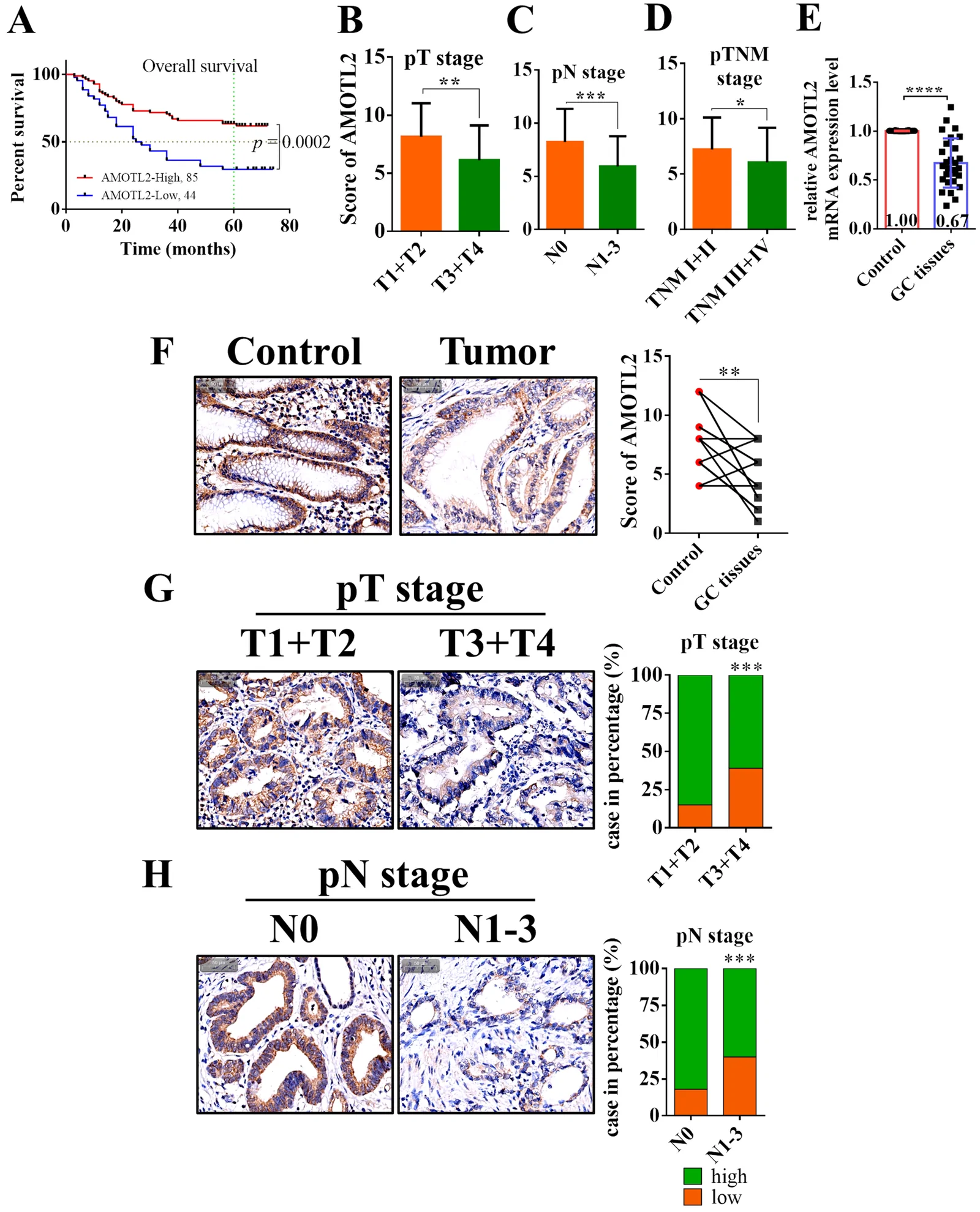
** Reduced AMOTL2 expression is associated with aggressive clinicopathological features and shorter survival.** Kaplan-Meier analysis of overall survival according to AMOTL2 IHC category in the first GC TMA (high, n = 85; low, 44) (A); AMOTL2 IHC scores according to tumor invasion (B), lymph-node status (C), and pTNM stage (D); AMOTL2 mRNA in 28 paired GC and adjacent non-tumor tissues (E, n = 28); representative IHC images and paired AMOTL2 scores in GC and adjacent non-tumor tissues (F, n = 14); distribution of high and low AMOTL2 expression according to T stage and lymph-node status (G-H). Data are presented as mean ± SD. The reported analyses used the log-rank (Mantel-Cox) test for overall survival analysis (A); the unpaired Student's t-test for B-D; the paired Student's t-test for E-F; and the chi-square test for G-H. *p < 0.05, **p < 0.01, ***p < 0.001, ****p < 0.0001.

AMOTL2 expression was evaluated in the first GC TMA employed in our previous study 49. Survival analysis showed that patients with low AMOTL2 expression had shorter overall survival than those with high expression (log-rank test, *p =* 0.0002; median survival time, 30 months vs. not reached; **Figure 1A**). Moreover, AMOTL2 IHC scores were lower in tumors with advanced T stage, lymph node metastasis, and higher TNM stage (**Figure 1B-D**). Consistently, in 28 paired samples, AMOTL2 mRNA levels were also reduced in GC tissues relative to adjacent non-tumor tissues (relative expression, 0.67 vs. 1, *p <* 0.0001; **Figure 1E**). Representative IHC images and paired scoring further showed lower AMOTL2 expression in tumor tissues (*p =* 0.0033, **Figure 1F**), and this low expression was more frequent in cases with advanced pT stage or lymph node metastatic cases (**Figure 1G-H**).

In the dichotomized IHC analysis, low AMOTL2 expression was associated with deeper tumor invasion (pT,* p =* 0.024) and lymph node metastasis (pN, *p =* 0.018) (**Table 1**). In univariate Cox regression analysis, pT, pN, pTNM stage, differentiation, and AMOTL2 expression were correlated with overall survival (all *p ≤* 0.001) (**Table 2**). In multivariate analysis, lymph node metastasis (*p =* 0.016) and AMOTL2 expression (*p =* 0.024) remained independently associated with survival. These data suggest that AMOTL2 is a candidate prognostic indicator, although independent validation is required.

### AMOTL2 inhibited GC cell proliferation, migration, and invasion *in vitro*

**Figure 2:**
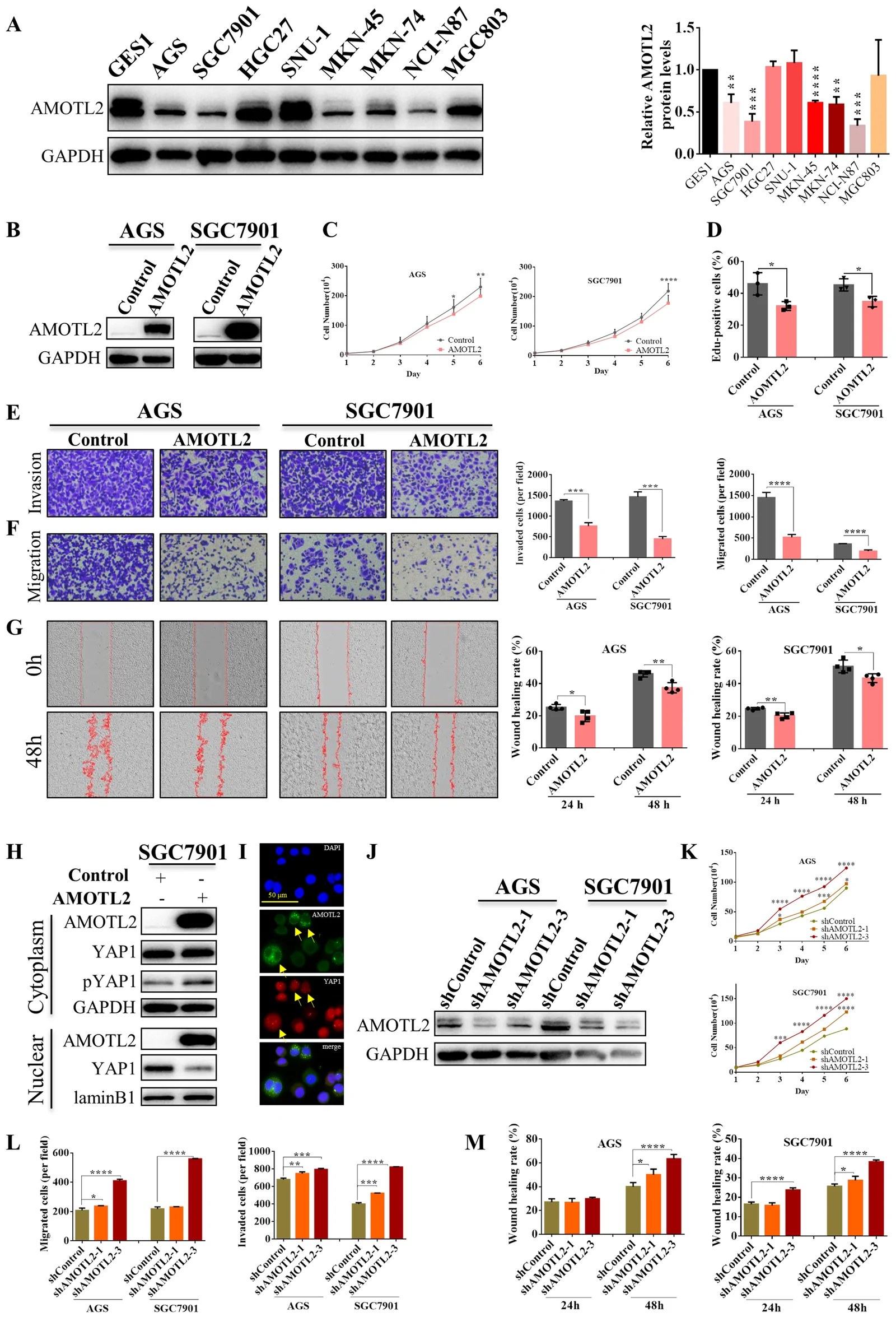
** AMOTL2 inhibits cell proliferation, migration, and invasion *in vitro*.** AMOTL2 protein abundance in GES-1 and GC cell lines (A, n = 3); validation of AMOTL2 overexpression in AGS and SGC7901 cells (B); cell-growth curves and EdU incorporation after AMOTL2 overexpression (C-D, n = 3); Transwell invasion (E, n = 3), migration (F, n = 3), and wound healing assays (G, n = 3); nuclear-cytoplasmic fractionation and immunofluorescence assessing Ser127 phosphorylation and nuclear accumulation of YAP1 in AMOTL2-overexpression cells and control cells (H-I); validation of AMOTL2 knockdown with two independent shRNAs (J); cell growth (K, n = 3), Transwell invasion/migration (L, n = 3), and wound healing capacity after AMOTL2 knockdown (M, n = 3). Data are presented as mean ± SD. The reported analyses used two-way ANOVA for cell growth curves (C, K); the unpaired Student's t-test for A, D-G, and L-M. *p < 0.05, **p < 0.01, ***p < 0.001, ****p < 0.0001.

AMOTL2 protein abundance was lower in most tested GC cell lines than in GES-1 cells (**Figure 2A**). AMOTL2 overexpression using Ubi-MCS-SV40-AMOTL2 lentivirus (**Figure 2B**) significantly suppressed cell proliferation, as shown by cell growth curve and EdU incorporation (AGS, 46% to 32%, *p =* 0.0325; SGC7901, 45% to 35%, *p =* 0.0222; **Figure 2C-D**). AMOTL2 overexpression also inhibited cell invasion and migration. The number of invaded cells decreased from 1365 to 762 in AGS cells (*p =* 0.0002), and from 1468 to 449 in SGC7901 cells (*p =* 0.0002) (**Figure 2E-F**). Wound healing assays showed a similar reduction in migration (**Figure 2G**). Nuclear-cytoplasmic fractionation and immunofluorescence further showed AMOTL2 promoted YAP1 Ser127 phosphorylation and inhibited nuclear YAP1 accumulation, consistent with reduced YAP activity (**Figure 2H-I**).

Additionally, AMOTL2 knockdown with two independent shRNAs (**Figure 2J**) enhanced cell proliferation, migration, and invasion (**Figure 2K-M**). In SGC7901 cells, AMOTL2 knockdown significantly increased the number of invaded cells from 400 to 823 (*p <* 0.0001), and the number of migrated cells from 218 to 558 (*p <* 0.0001) (**Figure 2L**). At 48 h, the wound healing rate also increased from 40.02% to 63.45% in AGS cells (*p <* 0.0001), and from 25.67% to 38.31% in SGC7901 cells (*p <* 0.0001) (**Figure 2M**). Collectively, these gain- and loss-of-function results indicate that AMOTL2 suppresses proliferative and metastatic phenotypes in tested cell models.

### AMOTL2 was inversely associated with VM and reduced tube formation

**Figure 3:**
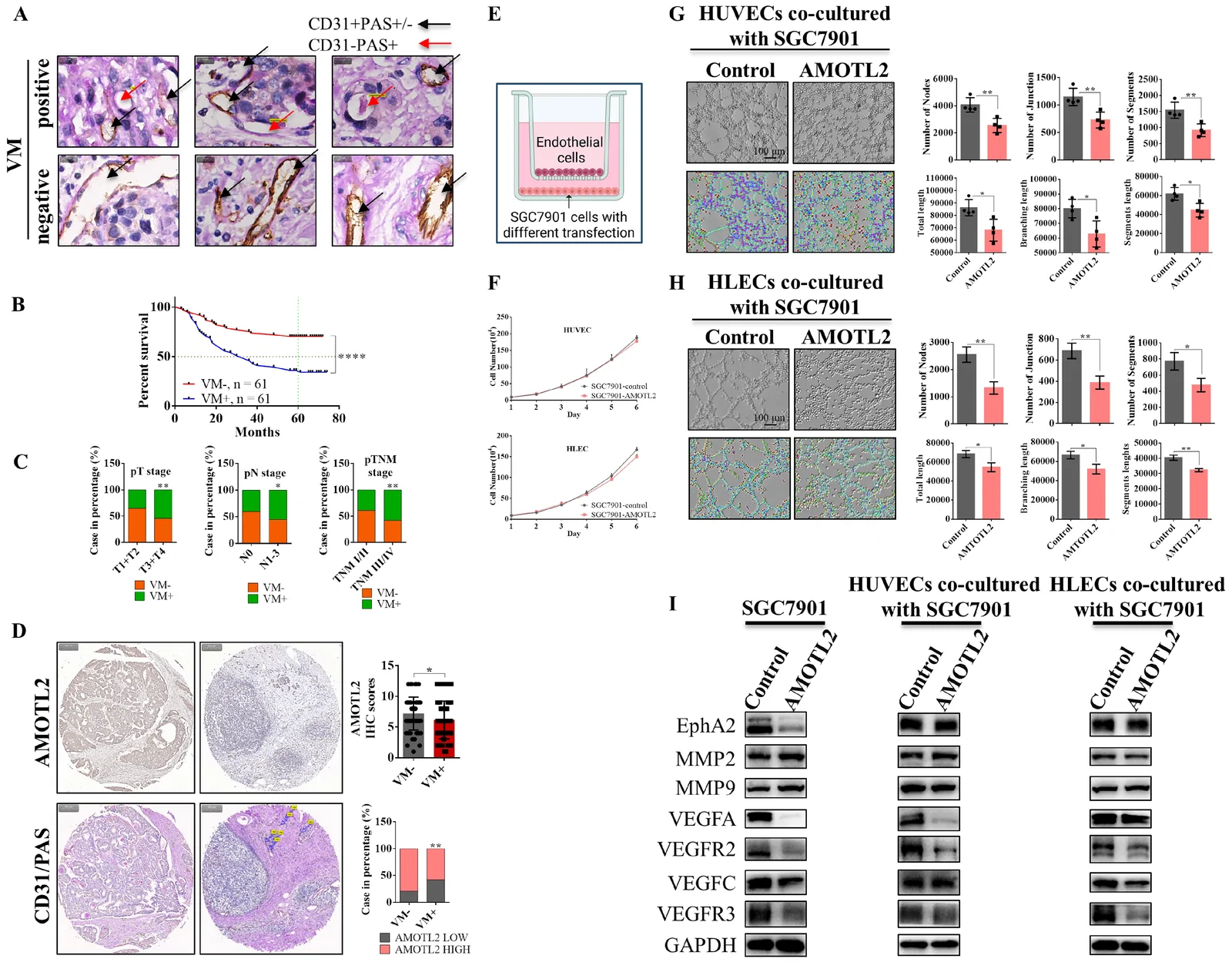
** AMOTL2 is inversely associated with VM and reduces tube formation.** Representative CD31/PAS double staining showing CD31^-^/PAS^+^ VM channels (red arrows) and CD31^+^ endothelial vessels (black arrows) (A); overall survival according to VM (B); association of VM with tumor invasion, lymph-node status, and pTNM stage (C); AMOTL2 IHC scores and expression according to VM status (D); schematic of SGC7901/endothelial cell Transwell co-culture system (E); HUVECs and HLECs growth curves during co-culture (F, n = 3), representative tube-formation images and quantitative results in HUVECs and HLECs co-cultured with SGC7901-AMOTL2 or control cells (G-H, n = 3); immunoblotting of angiogenic and lymphangiogenic proteins in tumor cells and co-cultured HUVECs and HLECs (I). Data are presented as mean ± SD. The reported tests were log-rank (Mantel-Cox) test (B); the chi-square test (C, lower panel of D); the unpaired Student's t-test (upper panel of D, G, and H), and two-way ANOVA (F). *p < 0.05, **p < 0.01, ***p < 0.001, ****p < 0.0001.

Vasculogenic mimicry (VM) was defined as CD31-negative/PAS-positive tumor cell channels (red arrows, CD31^-^/PAS^+^), whereas CD31-positive channels were classified as endothelial vessels (black arrows, CD31^+^) (**Figure 3A**). In a reanalysis of our previously reported cohort 49, VM-positive tumors were associated with shorter overall survival (median, 36 months vs. not reached; *p <* 0.0001), deeper tumor invasion (*p =* 0.0069), lymph-node metastasis (*p =* 0.0337), and advanced TNM stage (*p =* 0.0072) (**Figure 3B-C**). AMOTL2 IHC scores were lower in VM-positive than in VM-negative tumors (6.15 vs. 7.20; *p* = 0.0484), and low AMOTL2 expression was more frequent in VM-positive cases (*p =* 0.0014) (**Figure 3D**).

In the Transwell co-culture system, HUVECs and HLECs exposed to SGC7901-AMOTL2 cells exhibited impaired tube formation compared with those exposed to control cells, with significant reductions in nodes, junctions, segments, total length, branching length, and segment length (*p <* 0.05 for all), whereas cell growth curves were not detectably altered (**Figure 3E**-**H**). AMOTL2 overexpression downregulated EphA2, VEGFA, VEGFR2, VEGFC, and VEGFR3 in SGC7901 cells, whereas MMP2 and MMP9 remained unchanged. VEGFA and VEGFR2 were reduced in co-cultured HUVECs, and VEGFC and VEGFR3 were reduced in co-cultured HLECs (**Figure 3I**). Thus, AMOTL2 expression was inversely associated with VM in clinical tissues and attenuated angiogenic and lymphangiogenic tube-formation phenotypes in co-cultured endothelial cells.

### AMOTL2 overexpression suppressed tumor growth and lymph-node metastasis *in vivo*

**Figure 4:**
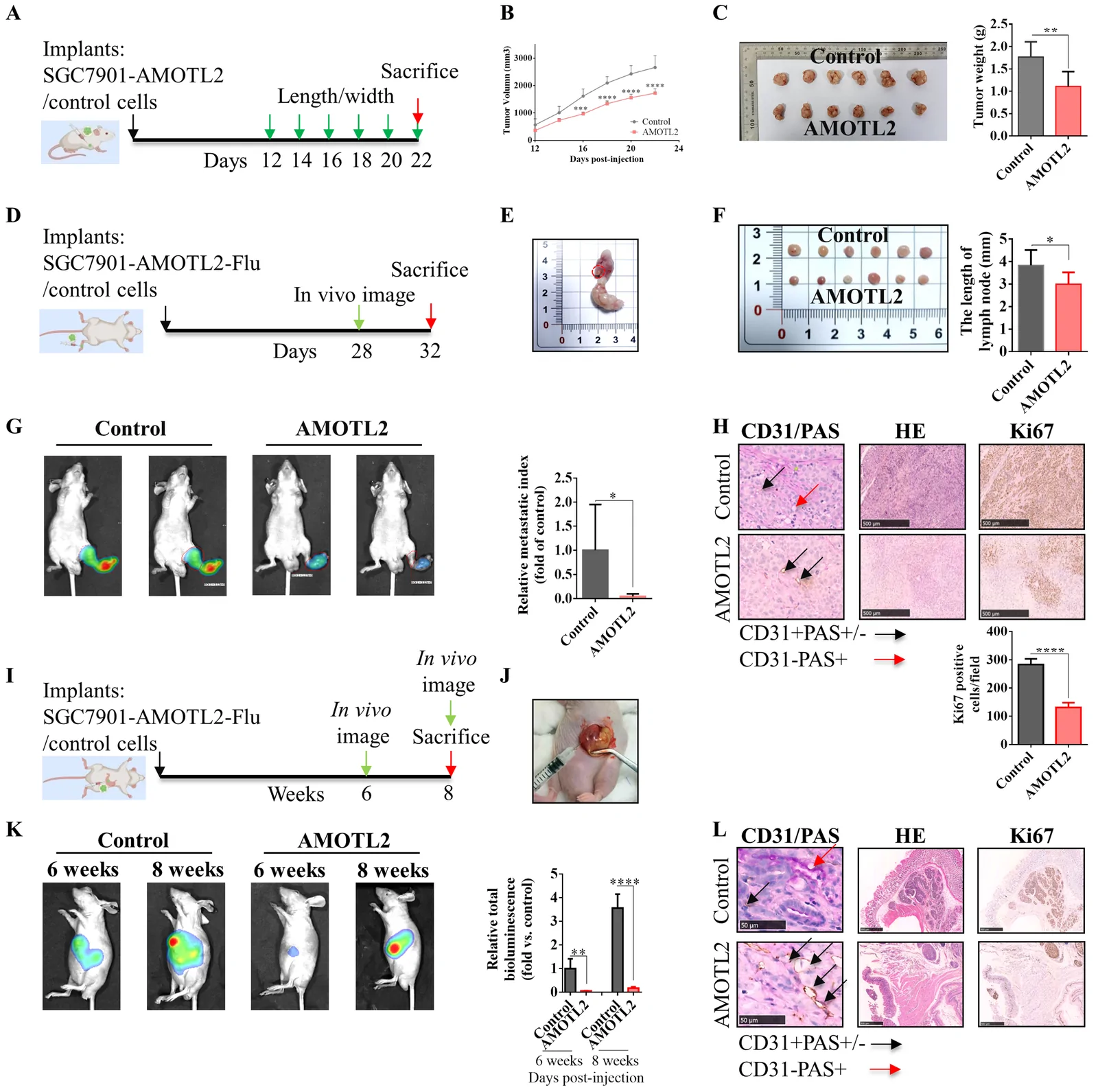
** AMOTL2 overexpression reduces xenograft growth and lymph-node dissemination**. Flowchart, growth curves, and terminal weights of subcutaneous xenografts generated with SGC7901-AMOTL2 or control cells (A-C, n = 6); Flowchart, representative specimens, the length of popliteal lymph node, the metastatic index (the ratio of bioluminescence signal in isolated lymph node to the total signal (lymph node and footpad)), and H&E, Ki67, and CD31/PAS staining (D-H, n = 6); panel G (left), images 1 and 2 are from the same control mouse, and images 3 and 4 from the same AMOTL2-overexpressing mouse. Images 1 and 3 show the total signal (lymph node and footpad), whereas images 2 and 4 represent the isolated popliteal lymph node signal; gastric orthotopic model, including flowchart, operative image, serial bioluminescence, and H&E, Ki67, and CD31/PAS staining (I-L, n = 4). Data are presented as mean ± SD. The reported analyses used two-way ANOVA for the subcutaneous growth curve (B); the unpaired Student's t-test for C, F, G, H, and K. *p < 0.05, **p < 0.01, ***p < 0.001, ****p < 0.0001.

The *in vivo* effects of AMOTL2 were evaluated using three xenograft models. In the subcutaneous xenograft model, AMOTL2-overexpressing tumors showed smaller tumor volumes and had lower weights compared with control tumors (**Figure 4A-C**). In the popliteal lymph node metastasis model, AMOTL2-overexpressing tumors exhibited smaller primary footpad tumors and reduced lymphatic metastasis to the popliteal lymph nodes, as evidenced by lymph node metastatic index (defined as the ratio of isolated popliteal lymph node signal to the total signal from both lymph node and footpad) and lymph node length (**Figure 4D-G**). Ki67 staining and CD31/PAS analysis of footpad tumors showed lower proliferative activity and fewer VM structures in AMOTL2 overexpression group (**Figure 4H**). In the orthotopic model, AMOTL2 overexpression suppressed orthotopic tumor growth, as shown by bioluminescence at the measured time points (**Figure 4I-K**). Histologic analyses showed lower Ki67 staining and fewer VM structures (**Figure 4L**). Collectively, three *in vivo* models provided complementary evidence corroborating the inhibitory role of AMOTL2 in xenograft growth and lymph-node dissemination.

### AMOTL2 suppressed the TGF-β/Smad pathway

**Figure 5:**
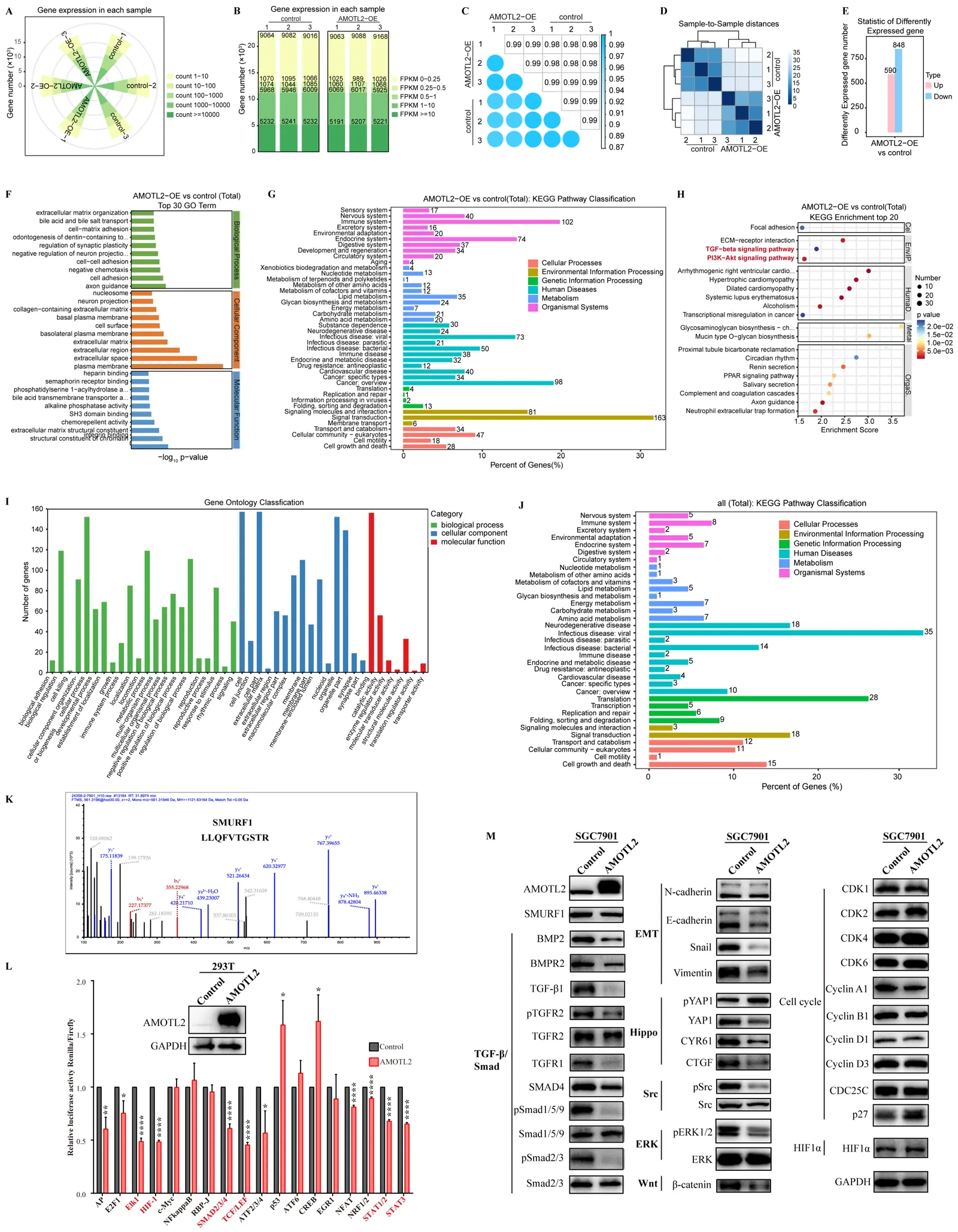
** AMOTL2 suppresses the TGF-β/Smad signaling and affects multiple signaling pathways.** mRNA sequencing overview comparing SGC7901-AMOTL2 and control cells, including differential-expression and sample-level analyses (A-E); GO and KEGG enrichment analyses (F-H); immunoprecipitation-mass spectrometry candidate AMOTL2-binding proteins, including SMURF1 (I-K); 18-pathway reporter screen after AMOTL2 overexpression (L, n = 3); immunoblotting of BMP/TGF-β-Smad, Hippo-YAP, EMT, ERK, Src, Wnt, and hypoxia-related proteins (M). Data are presented as mean ± SD. The reported analysis used the unpaired Student's t-test for L. *p < 0.05, **p < 0.01, ***p < 0.001, ****p < 0.0001.

mRNA sequencing of SGC7901-AMOTL2 and control cells identified 590 upregulated and 848 downregulated genes (**Figure 5A-E**). GO and KEGG enrichment analyses implicated cell adhesion, focal adhesion, ECM-receptor interaction, cytokine signaling, PI3K-AKT, and MAPK signaling (**Figure 5F-H**). Additionally, immunoprecipitation-mass spectrometry identified SMURF1 as a candidate AMOTL2-interacting protein (**Figure 5I-K**).

Pathway-reporter screening showed lower activity of several reporters after AMOTL2 overexpression, including reporters related to MAPK/ERK, hypoxia, TGF-β/Smad, Wnt, STAT1, and STAT3 signaling (**Figure 5L**; reporter sequences are provided in **Supplementary Table S4**).

Moreover, western blot analysis showed that AMOTL2 overexpression suppressed the TGF-β/Smad axis, as evidenced by reduced BMP2, BMPR2, TGF-β1, and TGF-β receptor I (TGFBR1), and Smad4 abundance, and decreased phosphorylation of TGFBR2, Smad1/5/9, and Smad2/3 (**Figure 5M**). In parallel, AMOTL2 promoted YAP1 Ser127 phosphorylation and reduced its downstream targets CYR61 and CTGF. EMT markers N-cadherin, Snail, and Vimentin were decreased, whereas E-cadherin was not detectably altered. Additionally, ERK and Src phosphorylation and β-catenin abundance were reduced, whereas the tested cell-cycle and hypoxia-associated proteins were minimally affected. Collectively, these results suggest that AMOTL2 broadly modulates multiple signaling pathways, with TGF-β/Smad signaling serving as a primary mediator, while concurrent suppression of ERK/Src and Wnt pathways may cooperatively reinforce its tumor-suppressive function.

### The Smad1/5/9 agonist SB4 partially reversed AMOTL2-mediated tumor suppression

**Figure 6:**
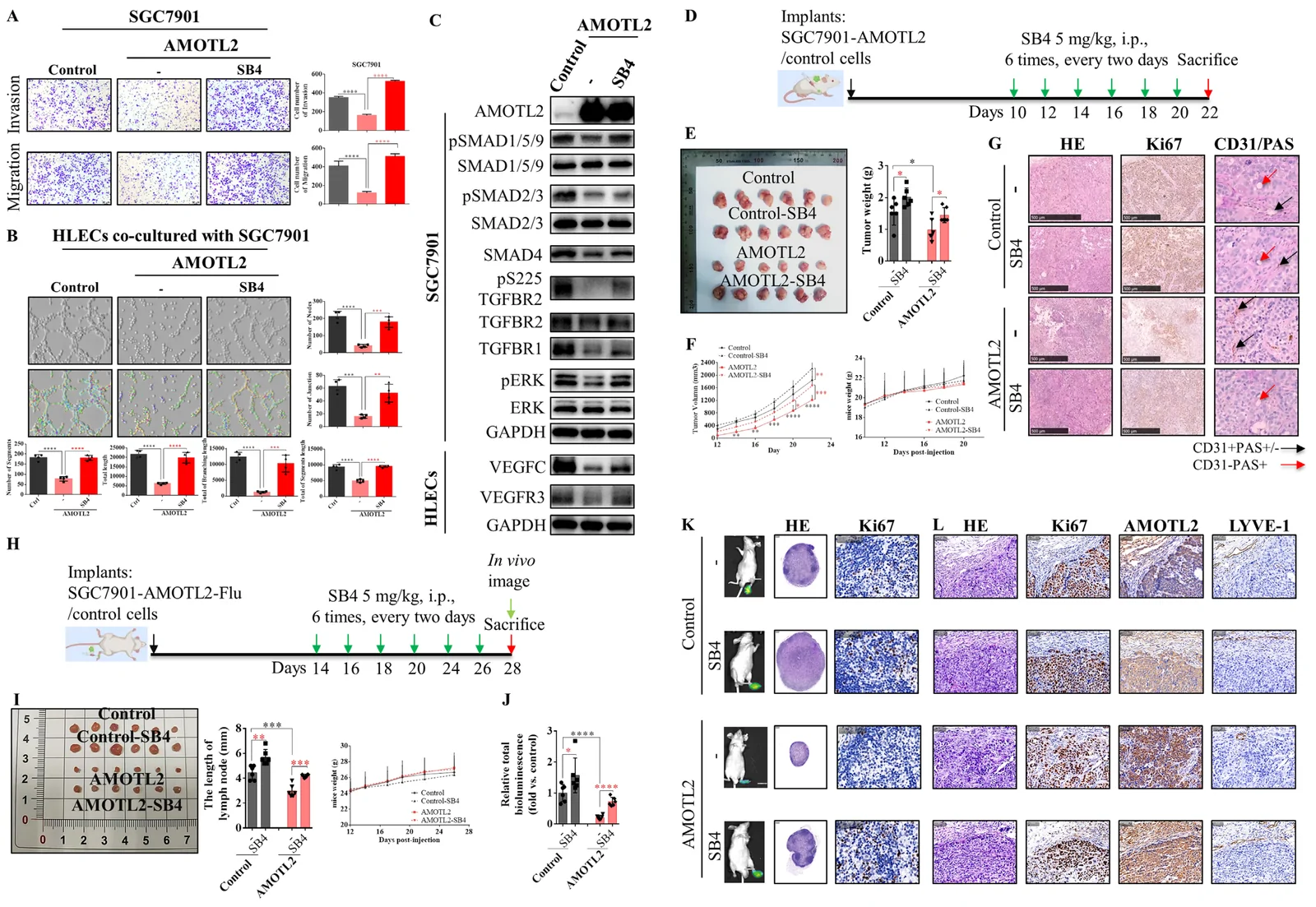
** The Smad1/5/9 agonist SB4 partially reverses AMOTL2-mediated tumor suppression.** Transwell migration and invasion after treatment with SB4 or vehicle in SGC7901-AMOTL2-overexpression setting (A, n = 3); HLECs tube formation upon co-culture with the indicated SGC7901 cells and treatments (B, n = 3); immunoblotting of Smad-pathway proteins in SGC7901 cells and VEGFC and VEGFR3 in co-cultured HLECs (C); the subcutaneous model, including flowchart, terminal tumor weight, tumor-growth curve, mice body weight, and H&E, Ki67, and CD31/PAS staining (D-G, n = 6); the popliteal lymph node metastasis model, including experiment workflow, lymph node length, total bioluminescence, and H&E staining, IHC for Ki67, AMOTL2, and LYVE-1 (H-L, n = 6). Black asterisks denote comparisons between AMOTL2 and control groups, red asterisks denote comparisons between SB4 and vehicle in the AMOTL2 setting (A, B, E, F, I, and J). Data are presented as mean ± SD. The reported analyses used the unpaired Student's t-test for A, B, E, I (middle panel), and J; and two-way ANOVA for the tumor growth and body weight curves in F and I (right panel). *p < 0.05, **p < 0.01, ***p < 0.001, ****p < 0.0001.

To investigate the functional contribution of Smad signaling, SGC7901-AMOTL2 cells were treated with Smad1/5/9 agonist SB4, a small-molecule BMP-signaling agonist that stabilizes or enhances Smad1/5/9 activation 52. SB4 treatment (1 μM, 24 h) largely restored cell migration, invasion, and HLEC tube formation in the AMOTL2-overexpression setting (**Figure 6A-B**). SB4 increased the phosphorylation of Smad1/5/9, whereas Smad2/3 phosphorylation was not clearly elevated. Moreover, SB4 increased VEGFC and VEGFR3 abundance in co-cultured HLECs (**Figure 6C**).

In the subcutaneous model, mice bearing SGC7901-AMOTL2 tumors were administered intraperitoneally with SB4 (5 mg/kg) every 2 days from day 10 to 20 (**Figure 6D**). SB4 largely increased tumor weight (*p =* 0.0204) and volume relative to vehicle-treated AMOTL2-overexpressing tumors (**Figure 6E-F**). Ki67 and CD31/PAS staining were also increased, consistent with partial restoration of proliferation and VM-associated structures (**Figure 6G**).

In the popliteal lymph node metastasis model, SB4 increased primary-tumor and lymph-node dissemination in the AMOTL2-overexpression setting, including popliteal lymph-node length (*p =* 0.0001) and total bioluminescence (*p <* 0.0001) (**Figure 6H-J**). H&E staining and IHC for Ki67 and LYVE-1 in footpad tumors consistently revealed increased proliferation and lymphatic-vessel density (**Figure 6K-L**). Furthermore, using a human-specific anti-Ki67 antibody that does not recognize murine cells in the popliteal lymph nodes, we observed that AMOTL2 overexpression reduced the proliferative index of the disseminated human gastric cancer cells, an effect that was reversed by SB4. Collectively, these pharmacologic rescue data indicate that Smad1/5/9 contributes to tumor-suppressive phenotypes of AMOTL2.

### SMURF1 interacted with AMOTL2 through its WW domains and promoted its ubiquitination and turnover

**Figure 7:**
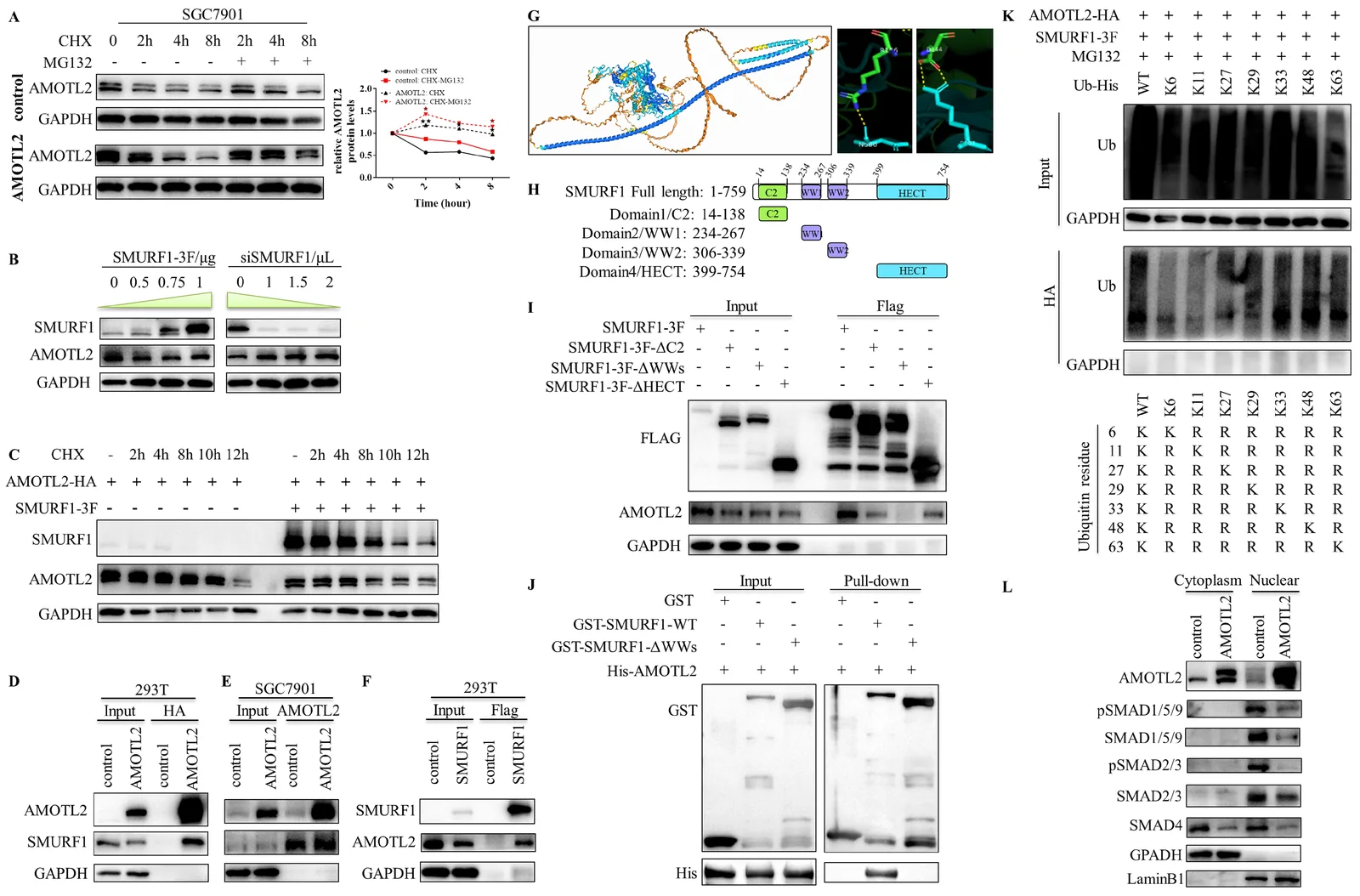
** SMURF1 interacts with AMOTL2 through its WW domains and promotes its ubiquitination and turnover.** Cycloheximide-chase analysis of AMOTL2 with or without MG132 (A, n = 3); AMOTL2 protein abundance after SMURF1 overexpression or knockdown (B); cycloheximide-chase analysis with or without SMURF1 overexpression (C); reciprocal co-immunoprecipitation of tagged proteins and endogenous AMOTL2-SMURF1 co-immunoprecipitation (D-F); AlphaFold3 prediction of a putative AMOTL2-SMURF1 complex (G); SMURF1 domain map and truncation analysis implicating the WW domains in AMOTL2 binding (H-I); GST pull-down with His-tagged AMOTL2, full-length SMURF1, a WW domains deletion construct, and GST control (J); cell-based ubiquitination assays using the indicated ubiquitin variants (K); nuclear-cytoplasmic distribution of phosphorylated and total Smad proteins after AMOTL2 overexpression (L). Black stars denote comparisons between SGC7901-AMOTL2 and control cells with sole CHX treatment, brick-red stars denote comparisons under combined treatment with CHX and MG132 (A). Data are presented as mean ± SD. The reported analysis used two-way ANOVA in A. *p < 0.05, **p < 0.01, ***p < 0.001, ****p < 0.0001.

Cycloheximide-chase and MG132 assays showed that the decrease in AMOTL2 protein levels was attenuated by proteasome inhibition, consistent with proteasome-dependent turnover (**Figure 7A**). SMURF1 overexpression reduced AMOTL2 protein abundance in a dose-dependent manner, whereas SMURF1 knockdown increased its abundance (**Figure 7B**). SMURF1 also accelerated the decrease in AMOTL2 protein levels in CHX chase assays (**Figure 7C**).

Reciprocal co-immunoprecipitation of tagged proteins in HEK293T cells and endogenous co-immunoprecipitation supported an interaction between AMOTL2 and SMURF1 (**Figures 7D**-**F**). AlphaFold3 complex prediction provided a putative structural model, and truncation analysis revealed that the WW domains of SMURF1 are critical for its binding to AMOTL2 (**Figure 7G-I**). GST pull-down further supported the direct *in vitro* interaction and demonstrated that the WW domains are required for this binding (**Figure 7J**). Cell-based ubiquitination assays showed increased K33-, K48-, and K63-linked ubiquitin signals associated with AMOTL2 in the presence of SMURF1 (**Figure 7K**). Nuclear-cytoplasmic fractionation additionally revealed that AMOTL2 reduces nuclear accumulation of phosphorylated and total Smad1/5/9 and Smad2/3, as well as total Smad (**Figure 7L**).

### SMURF1 was associated with adverse clinical features and promoted malignant phenotypes

**Figure 8:**
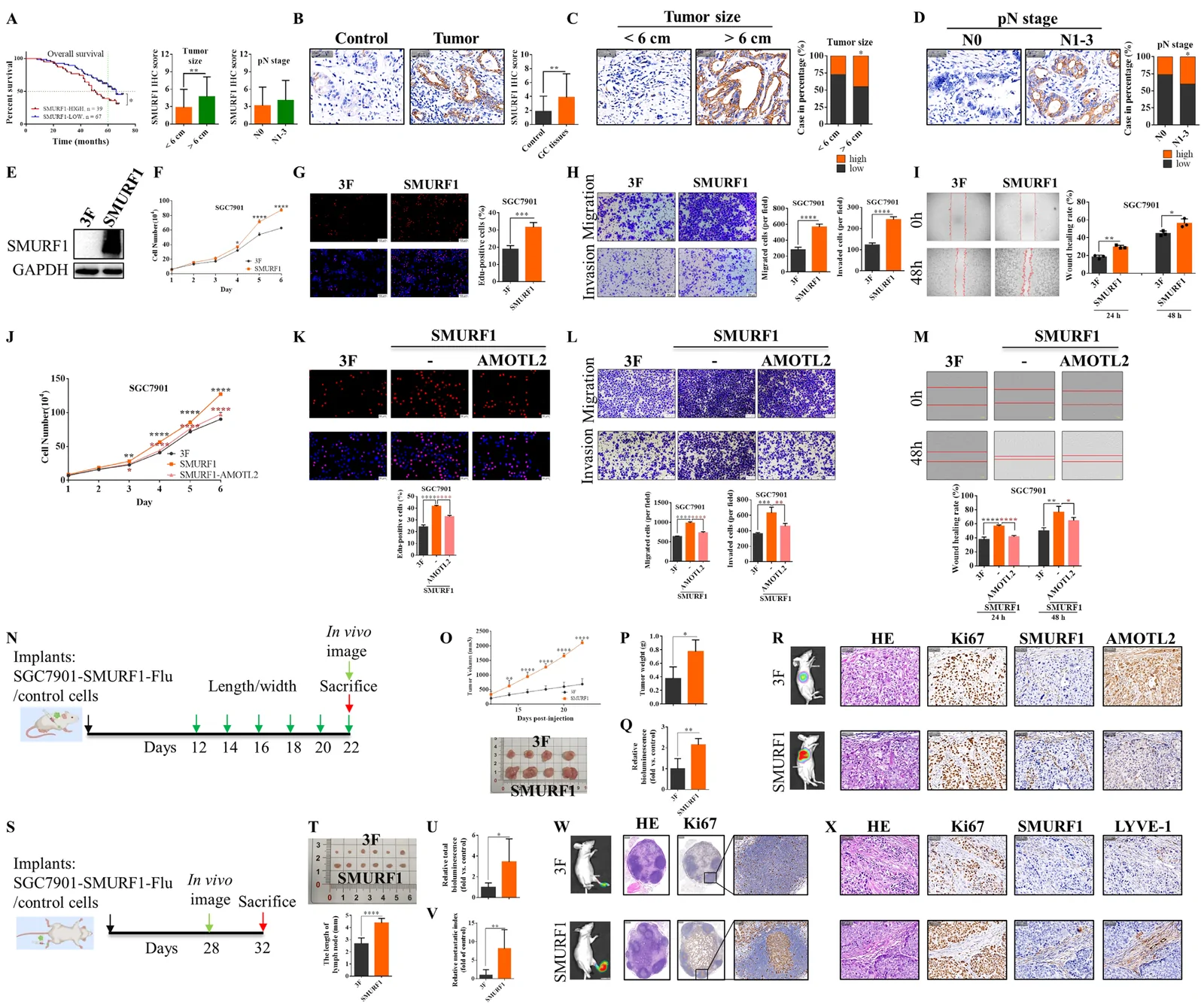
** SMURF1 is associated with adverse clinical features and promotes malignant phenotypes in cell and mouse models.** Overall survival, tumor-size correlation, tumor/non-tumor IHC, and lymph-node-status association in the second GC TMA (A-D); validation of SMURF1 overexpression and effects on cell proliferation, EdU incorporation, Transwell migration and invasion, and wound healing (E-I, n = 3); *in vitro* rescue by AMOTL2 co-expression (J-M, n = 3); the subcutaneous xenograft model, including the flowchart, tumor growth, terminal tumor weight, total bioluminescence, H&E staining, and IHC for Ki67, SMURF1, and AMOTL2 (N-R, n = 4); the popliteal lymph node metastasis model, including the flowchart, lymph-node length, total tumor burden and metastatic index in lymph node, H&E staining, and IHC for Ki67, SMURF1, and LYVE-1 (S-X, n = 6). Black asterisks denote comparisons between SMURF1 and control cells; brick-red asterisks denote comparisons with and without AMOTL2 co-overexpression in SMURF1-overexpressing setting (J-M). Data are presented as mean ± SD. The reported analyses used the log-rank (Mantel-Cox) test for left panel of A; the unpaired Student's t-test for the middle and right panels of A, B, G-I, K-M, P, Q, and T-V; the chi-square test for C and D; and two-way ANOVA for growth curves in F, J, and O. *p < 0.05, **p < 0.01, ***p < 0.001, ****p < 0.0001

In the second GC TMA, elevated SMURF1 expression was associated with shorter overall survival (median, 51 vs. 66 months; *p =* 0.0418) and larger tumor size (*p =* 0.0039) (**Figure 8A**). SMURF1 IHC scores were higher in GC tissues than in adjacent non-tumor tissues (3.95 vs. 1.86; *p =* 0.0085), and this high expression was more frequent in cases with larger size tumor (*p =* 0.0120) or lymph node metastatic cases (*p =* 0.0353) (**Figure 8B-D**). In the dichotomized analysis, SMURF1 expression was positively associated with larger tumor size (*p =* 0.045) (**Supplementary Table S1**). The reported multivariate model identified SMURF1 expression (*p =* 0.041) and lymph node metastasis (*p =* 0.025) as independent prognostic factors (**Supplementary Table S2**).

SMURF1 overexpression in GC cell lines enhanced cell proliferation, EdU incorporation, Transwell invasion and migration, and wound healing (all *p <* 0.05) (**Figure 8E-I**). Co-expression of AMOTL2 suppressed these SMURF1-driven phenotypes (**Figure 8J-M**). In the subcutaneous xenografts, SMURF1 overexpression resulted in larger tumor volume (*p <* 0.0001), higher terminal tumor weight (*p =* 0.0162), stronger bioluminescence (*p =* 0.0066), and increased Ki67 staining, along with reduced AMOTL2 staining (**Figure 8N-R**). In the popliteal lymph node metastasis model, SMURF1 overexpression increased total tumor burden (lymph node plus footpad) and metastatic index in lymph node (*p =* 0.0247 and *p =* 0.0080, respectively), as well as popliteal lymph node length (4.38 vs. 2.67 mm, *p <* 0.0001) (**Figure 8S-V**). H&E, Ki67, and LYVE-1 staining in the primary footpad tumors consistently indicated increased proliferation and lymphatic-vessel density (**Figure 8W-X**). In the popliteal lymph nodes, immunostaining with a human-specific anti-Ki67 antibody that does not cross-react with murine cells further demonstrated that SMURF1 overexpression significantly increased the proliferative index of the metastatic human gastric cancer cells. Notably, we observed that the distribution patterns of SMURF1 in xenograft tumors, including both subcutaneous and footpad models, diverged substantially from those detected in human GC clinical tissues. Collectively, these results establish SMURF1 as an oncogenic driver in GC cell lines and mouse models and show that AMOTL2 restoration can counteract SMURF1-driven phenotypes *in vitro*.

## Discussion

In this study, we define AMOTL2 as a novel tumor suppressor in GC. AMOTL2 expression was clinically reduced in GC tissues, and its low expression was associated with deeper invasion, lymph-node metastasis, and shorter overall survival. Functionally, AMOTL2 suppressed cell proliferation, migration, and invasion, and impaired endothelial and lymphatic-endothelial tube formation, accompanied by downregulation of VEGFA/VEGFR2 and VEGFC/VEGFR3. Xenograft models further demonstrated that AMOTL2 overexpression reduced tumor growth and lymphatic dissemination. Mechanistically, our findings indicate that AMOTL2 attenuates BMP/TGF-β/Smad signaling, while SMURF1 interacts with AMOTL2 and promotes its ubiquitination and turnover. Furthermore, using a human-specific anti-Ki67 antibody, we demonstrated that AMOTL2 overexpression reduced the proliferative index of metastatic human GC cells in popliteal lymph nodes. This suppressive effect was abrogated by SB4-mediated Smad1/5/9 activation, providing *in vivo* evidence that AMOTL2 inhibits lymph node colonization partially through Smad signaling, especially Smad1/5/9 branch.

Other angiomotin-family members AMOT and AMOTL1 have been studied in GC. Reduced AMOT-p130 is associated with aggressive features, and restoration of AMOT-p130 can limit cell migration and invasion by inhibiting EMT 53, 54. AMOT knockdown impedes cell migration by dissociating YAP from ZO-1, affecting tight-junction organization and migration 55. In contrast, increased AMOTL1 is linked to poor prognosis, and enhances YAP1 stability to promote gastric oncogenesis 56. These previous studies underscore that individual angiomotin-family proteins have distinct and context-dependent functions. In addition, we have published a review detailing the cellular localization and specific effects of AMOTL2 in tumor and normal cells 57. Thus, this study is the first to demonstrate the tumor-suppressive effects of AMOTL2 in GC progression, thereby expanding the current understanding of this family.

The relationship between AMOTL2 and YAP1 signaling is likewise context dependent. Multiple lines of evidence support AMOTL2 as a YAP1 suppressor: AMOTL2 can facilitate LATS2 and YAP phosphorylation 5, 11, 58, and its mono-ubiquitination (K347/K408) is essential for this process 9, 10. In airway smooth muscle cells and zebrafish, AMOTL2 also inhibits YAP1-driven proliferative activity 59, 60. In endocytic trafficking, the competition of AMOTL2 with Endotubin can regulate YAP localization 61. In contrast, AMOTL2 enhances YAP nuclear accumulation by stabilizing YAP1 in muscle stem cells 62. Our results demonstrate that AMOTL2 increased YAP1 phosphorylation at Ser127, reduced its nuclear accumulation, and decreased CYR61 and CTGF expression, consistent with inhibition of YAP1.

Beyond YAP1, AMOTL2 also participates in other multiple signaling pathways. Previous studies have shown that AMOTL2 can limit AKT membrane recruitment, and modulate ERK and Wnt/β-catenin signaling 6, 16, 63. Conversely, AMOTL2 has also been reported to exert pro-tumorigenic functions through promoting MAPK/ERK and p38 activation 17-19. Here, we found that AMOTL2 reduced BMP2/BMPR2 and TGF-β1/TGFBR2 signaling, decreased phosphorylation of both Smad1/5/9 and Smad2/3, reduced Smad4 abundance, and disrupted Smad nuclear accumulation. Furthermore, pharmacological rescue with SB4 substantially reversed several AMOTL2-associated suppressive phenotypes, emphasizing the functional significance of Smad1/5/9 pathway in AMOTL2-mediated tumor suppression.

The interaction studies identify SMURF1 as a regulator of AMOTL2 protein stability. Reciprocal and endogenous co-immunoprecipitation, WW domains mapping, and GST pull-down assays support their physical interaction, whereas cycloheximide-chase, MG132, and ubiquitination assays supported SMURF1-mediated ubiquitination and proteasomal degradation of AMOTL2. K48-linked ubiquitination provides a plausible degradative signal, whereas the functional roles of K33- and K63-linked signals remain to be defined. In addition, consistent with most previous studies, SMURF1 was elevated in GC tissues and correlated with shorter overall survival; its overexpression promoted malignant phenotypes *in vitro* and *in vivo*, effects that were effectively counteracted by AMOTL2 restoration. Notably, human-specific Ki67 IHC revealed SMURF1 overexpression significantly increased the proliferative index of metastatic human gastric cancer cells in popliteal lymph nodes, demonstrating its pro-metastatic function in lymph node colonization.

SMURF1 is recognized as a negative regulator of TGF-β/BMP signaling through degradation of R-Smads and receptor complexes 21, 23, 24, 64. In GC, SMURF1 is significantly increased and predicts poor prognosis 25, 27, 39, 42, promoting tumor growth and metastasis through PI3K/AKT and MEK/ERK signaling 25, 42. SMURF1 expression can be also suppressed by tumor-suppressive microRNAs such as miR-1254 and miR-424 27, 39, 40.

Although the observations that both SMURF1 and its substrate Smads promote GC progression appear paradoxical, this coexistence is not necessarily contradictory. Several non-mutually exclusive mechanisms may explain this contradiction. First, SMURF1 drives GC primarily through ubiquitination of non-Smad substrates, including PTEN, Axin2, and GSTM2 65-67, and, as shown here, AMOTL2. Therefore, the observed biological effects may reflect a dynamic balance among the substrates of SMURF1, rather than uniform suppression of Smad signaling. Second, SMURF1 modulates non-canonical, Smad-independent pathways, for instance, it activates the PI3K/AKT pathway by regulating DAB2IP expression, thereby driving malignant phenotypes in GC cells 25. Third, TGF-β/BMP signaling itself is highly context-dependent and shifts from tumor-growth restraint to promotion of invasion, metastasis, and immune evasion during tumor progression. In GC, Smad2/3 and Smad1/5/9 promote tumor progression through multiple signaling cascades, including the TGF-β2/Smad2/3/NDRG1 axis 43 and the BMP4/Smad1/5/9/ID1 pathway 48, highlighting the multifaceted roles of Smad signaling in GC malignancy. Thus, SMURF1 drives GC not primarily through degradation of Smads, but rather via ubiquitination of non-Smad substrates, engagement of non-canonical pathways, and modulation of TGF-β/BMP signaling.

However, several limitations should be acknowledged. First, AMOTL2 and SMURF1 were assessed in the different clinical cohort since the original TMA sections were unavailable. Second, the specific amino acid residues within the WW domains of SMURF1 that are essential for AMOTL2 binding remain unidentified, requiring future structural and mutational studies. Third, the discrepancy in SMURF1 distribution patterns across different tissues may arise from multiple factors, encompassing fundamental differences in the tumor microenvironment, interspecies physiological variation, and adaptive molecular changes conferred by ectopic implantation. Fourth, sample sizes in the gastric orthotopic tumor model and the subcutaneous SMURF1 overexpression model were limited (n = 4/group) in accordance with the 3R principles; nevertheless, the findings were consistent with the results obtained from other models. Fifth, the underlying mechanism by which AMOTL2 reduces BMP2 and TGF-β1 expression remains unknown.

In conclusion, this study defines AMOTL2 as a novel suppressor of GC with potential prognostic value that inhibits tumor progression and lymphatic dissemination. AMOTL2 attenuates the TGF-β/Smad signaling pathway through reducing BMP2/TGF-β1 expression and downstream Smads activation. In addition, SMURF1 bound AMOTL2 and promoted its ubiquitination and turnover. We propose the SMURF1-AMOTL2-Smad regulatory axis may serve as a critical modulator of GC progression, suggesting its prognostic relevance and therapeutic target potential, which require further validation in functional studies.

## Supplementary Material

Supplementary tables.

## Figures and Tables

**Table 1:** Association between AMOTL2 expression (0-4 vs. 6-12) and the clinicopathological parameters in the GC cohort.

Clinicopathological parameters	Total	AMOTL2		χ²	*p* value
		High expression (6-12)	Low expression (0-4)		
Gender				0.488	0.485
Male	101	65	36		
Female	28	20	8		
Age (years)				1.680	0.195
< 60	63	45	18		
≥ 60	66	40	26		
Tumor location				0.474	0.495
Upper	67	46	21		
Middle/lower	62	39	23		
Tumor size (cm)				0.017	0.896
< 6	86	57	29		
≥ 6	43	28	15		
Tumor differentiation				0.017	0.897
Well/moderate	42	28	14		
Poor/undifferentiated	87	57	30		
Tumor invasion depth					
T1/T2	26	22	4	5.080	0.024
T3/T4	103	63	40		
Lymph node metastasis					
N0	34	28	6	5.567	0.018
N1-3	95	57	38		
pTNM stage				1.350	0.245
I/II	53	38	15		
III/IV	76	47	29		

**Table 2:** Univariate and multivariate analyses assessing the correlation between the clinicopathological features, AMOTL2 expression, and overall survival.

Multivariate analysis	Univariate analysis		Multivariate analysis	
	HR (95%CI)	*p* value	HR (95%CI)	*p* value
Gender (male vs. female)	1.256 (0.655~2.410)	0.493		
Age (≥ 60 vs. < 60 years)	1.089 (0.664~1.786)	0.735		
Tumor location (upper vs. middle/lower)	1.137 (0.691~1.869)	0.613		
Tumor size (≥ 6 vs. < 6 cm)	1.627 (0.984~2.691)	0.058		
Differentiation (poor/undifferentiated vs. well/moderate)	4.949 (2.352~10.415)	< 0.001		
Tumor invasion depth (T3/T4 vs.T1/T2)	11.093 (2.709~45.432)	< 0.001		
Lymph node metastasis (N1-3 vs. N0)	3.684 (1.677~8.092)	0.001	2.698 (1.203~6.053)	0.016
pTNM stage (III/IV vs. I/II)	6.800 (3.343~13.830)	< 0.001		
AMOTL2 expression (high vs. low)	0.412 (0.251~0.677)	< 0.001	0.556 (0.333~0.927)	0.024

## Data Availability

Data from the current study are available from the corresponding author on reasonable request.
