# Supplementary material for: AMOTL2 inhibits lymph node metastasis in gastric cancer by suppressing TGF-β/Smad signaling and is destabilized by the E3 ubiquitin ligase SMURF1
Source: Int J Biol Sci. 2026 Aug 11;22(13):7191–208. doi: 10.7150/ijbs.135795 (PMC13540700; doi:10.7150/ijbs.135795)
Supplement: Supplementary file 1 [file ijbsv22p7191s1.pdf]

Table S1. Association between SMURF1 expression (0-4 vs. 6-12) and the clinicopathological parameters in the GC cohort.

Clinicopathological parameters	Total	SMURF1		χ^2	*p* value
		High expression (>4)	Low expression (≤4)		
Gender				1.316	0.251
Male	70	25	45		
Female	36	17	19		
Age (years)				1.150	0.284
< 60	42	14	28		
≥ 60	64	28	36		
Tumor location				0.095	0.758
Upper	70	27	43		
Middle/lower	36	15	21		
Tumor Size (cm)				**4.009**	**0.045**
< 6	48	14	34		
≥ 6	58	28	30		
Tumor metastasis					
T1/T2	28	11	17	0.002	0.966
T3/T4	78	31	47		
Tumor differentiation					
Well/moderate	41	17	24	0.095	0.758
Poor/undifferentiated	65	25	40		
Lymph node metastasis				0.288	0.592
N0	23	8	15		
N1-3	83	34	49		
pTNM stage				0.051	0.822
I/II	34	14	20		
III/IV	72	28	44		

Table S2. Univariate and multivariate analyses assessing the correlation between the clinicopathological features, SMURF1 expression, and overall survival.

Clinicopathological parameters	Univariate analysis		Multivariate analysis	
	HR (95%CI)	*p* value	HR (95%CI)	*p* value
Gender (male vs. female)	1.099 (0.617-1.957)	0.750		
Age (≥ 60 vs. < 60 years)	1.272 (0.732-2.212)	0.393		
Tumor location (upper vs. middle/lower)	1.153 (0.643-2.068)	0.634		
Tumor size (≥ 6 vs. < 6 cm)	0.682 (0.400~1.164)	0.161		
Tumor invasion depth (T3/T4 vs. T1/T2)	1.578 (0.827~3.008)	0.166		
Differentiation (poor/undifferentiated vs. well/moderate)	1.648 (0.918~2.957)	0.094		
Lymph node metastasis (N1-3 vs. N0)	**3.713 (1.474~9.353)**	**0.005**	**3.427 (1.165~10.088)**	**0.025**
pTNM stage (III/IV vs. I/II)	1.967 (1.034~3.739)	0.039		
SMURF1 expression (high vs. low)	**1.849 (1.081~3.162)**	**0.025**	**1.754 (1.023~3.008)**	**0.041**

Table S3. Primary and secondary antibodies.

Antibodies	Supplier	Catalog number	Dilution
**AMOTL2**	ABclonal	A16723	1:1000 (WB); 1:200 (IHC)
**SMURF1**	Abcam	ab300408	1:1000 (WB)
	Sigma	HPA055245	1:100 (IHC)
**CD31**	Abcam	ab28364	1:50 (IHC)
**GAPDH**	Sangon Biotech	D110016	1:1000
**HA-tag**	ABclonal	AE008	1:1000
**Flag-tag**	ABclonal	AE005	1:1000
**His-tag**	ABclonal	AE003	1:1000
**GST-tag**	ABclonal	AE001	1:1000
**YAP1**	ABclonal	A1002	1:1000
**CYR61**	ABclonal	A1111	1:1000
**CTGF**	ABclonal	A11067	1:1000
**Phospho-YAP1 (S127)**	Abcam	ab76252	1:1000
**Lamin B1**	ABclonal	A1910	1:1000
**EphA2**	ABclonal	A23177	1:1000
**VEGFA**	Proteintech	19003-1-AP	1:1000
**VEGF Receptor 2**	CST	2479	1:1000
**VEGFC**	ABclonal	A23163	1:1000
**VEGF Receptor 3**	ABclonal	A5603	1:1000
**MMP2**	Proteintech	10373-2-AP	1:1000
**MMP9**	Proteintech	10375-2-AP	1:1000
**HIF1α**	Proteintech	20960-1-AP	1:1000
**BMP2**	ABclonal	A0231	1:1000
**BMPR2**	ABclonal	A5666	1:1000
**TGF-β1**	ABclonal	A25313	1:1000
**Smad2/3**	ABclonal	A18674	1:1000
**Phospho-Smad2 (S465/S467) + Smad3 (S423/S425)**	ABclonal	AP1343	1:1000
**Smad1/5/9**	ABclonal	A23208	1:1000
**Phospho-Smad1 (S463/S465)+Smad5 (S463/S465)+Smad9 (S465/S467)**	ABclonal	AP1518	1:1000
**Smad4**	ABclonal	A19116	1:1000
**TGF-β Receptor I (TGFBR1)**	ABclonal	A0708	1:1000
**TGF-β Receptor II (TGFBR2)**	ABclonal	A1415	1:1000
**Phospho-TGF-β Receptor II (S225)**	Abcam	ab183037	1:2000
**E-cadherin**	Proteintech	20874-1-AP	1:1000
**N-cadherin**	ABclonal	A19083	1:1000
**Snail**	ABclonal	A11794	1:1000

**Vimentin**	ABclonal	A19607	1:1000
**CDK1**	MCE	HY-P80611	1:1000
**CDK2**	MCE	HY-P80076	1:1000
**CDK4**	Abcam	ab108357	1:1000
**CDK6**	Abcam	ab124821	1:1000
**Cyclin A1**	MCE	HY-P80631	1:1000
**Cyclin B1**	MCE	HY-P80632	1:1000
**Cyclin D1**	Abcam	ab134175	1:1000
**CDC25C**	Abcam	ab32444	1:1000
**p27**	Abcam	ab32034	1:1000
**β-catenin**	Zen-bio	R22820	1:1000
**Src**	CST	2123T	1:1000
**Phospho-Src (Y416)**	CST	6943T	1:1000
**ERK1/2**	Proteintech	51068-1-AP	1:1000
**Phospho-ERK1/2 (T202/Y204)**	Proteintech	28733-1-AP	1:1000
**ubiquitin**	Proteintech	10201-2-AP	1:1000
**Ki67**	CST	12202S	1:200 (IHC)
**Ki67**	Maxim	MAB-0672	1:100 (IHC)
**LYVE-1**	Abcam	ab219556	1:1000 (WB);<br>1:100 (IHC)
**Goat Anti-Rabbit**	Sangon Biotech	D110058	1:5000
**Goat Anti-mouse**	Sangon Biotech	D110087	1:5000
**Fc-specific secondary antibodies**	Abmart	M21008S	1:5000

Table S4. Core sequences of 18 luciferase reporters.

	Signaling pathways	Transcription factors	Response element
**1**	**MAPK/JNK**	AP1	TGACTCATGACTAA
**2**	**Cell cycle**	E2F1	TTTGGCGCGTAATTTGGCGCGTAATTTGGCGCGTAA
**3**	**MAPK/ERK**	ELK1	TTTGCAAAATGCAGGAATTGTTTTCACAGTACCGGAAGT
**4**	**Hypoxia**	HIF1	GTGACTACGTGCTGCCTAGTTACGTGC
**5**	**c-Myc**	Myc	CACGTGCACATGG
**6**	**NF-κB**	NF-κB	GGGAATTTCCGGGGACTTTCCCGGGAATTTGCGGGGGACTTTCCGGGAATTTGGGAATTTCCCGGGGATTCCCCGGGGAATCCCC
**7**	**Notch**	RBP-Jκ	CGTGGGAAAGTGGGAACGGCATTGTAGCG
**8**	**TGF-β1**	Smad	GTCTAGACAGCCAGACA
**9**	**Wnt**	TCF/LEF	AGATCAAAGGGGGTACCTTTGATCTT
**10**	**p53**	p53	GAACATGTCTAAGCATGCTGTGCCTTGCCTGGACTTGCCTGGACATGCCCGGGCATGT
**11**	**Amino Acid deprivation**	ATF2/3/4	ATGAGGTCATATGACGTCATAACATTGCATCATCCCCGCAACATTGCATCATCCCCGCATGATGCAATATGATGCAAT
**12**	**ATF6**	ATF6	TGATGACGTGGCATTCATCGAGACAGGTGCTGACGTGGCA
**13**	**cAMP/PKA**	CREB	TGATGTCAGCACCAGACAGTGACGTCATGGCCGTCATGACGTCACCCCATTGACGTCAATGGGAGAACTGATGTCACGAGGATATTGACGTCATGACGTCATGACGTCATGACGTCACAAGATCTG
**14**	**Early Growth Response**	EGR1	CGCCCCCGCGACGCCCACGC
**15**	**PKC/Ca2+**	NFAT	GGAGGAAAAACTGTTTCATACAGAAGGCGTATTTTCCAAATGGAAAAT
**16**	**Antioxidant Response**	Nrf1/2	ATGACTCAGCATGCGCACGCGCGGGTACCTCACAGTGACTCAGCATTTAAACACAGTGACTCAATGACTCAGCATGCGCACGCGCGGCATTTAAACACAGTGACTCAGCATTAAAGAGCTCGCTAGC
**17**	**Interferon gamma /Type I Interferon**	STAT1/2	GGAAAACGAAACTGCATTTCCCGGAAACCATGTTATGCATATTCCTGTAAGTGTTTCCAGGAAATCAGTTTCTTTTCC
**18**	**STAT3**	STAT3	CTTCTGGGAAATGCTTCCCGAACGTATTTCCCGTAAATCGTCGAAGCTTCCTTCTGGGAAA
